# In Vitro Replication of Human Norovirus

**DOI:** 10.3390/v11060547

**Published:** 2019-06-12

**Authors:** Sutonuka Bhar, Melissa K. Jones

**Affiliations:** Microbiology and Cell Science Department, University of Florida, Gainesville, FL 32611, USA; sutonuka.bhar@ufl.edu

**Keywords:** human norovirus, murine norovirus, human intestinal enteroids, norovirus replication, in vitro culture models

## Abstract

Human norovirus (HuNoV) infection is a major cause of gastroenteritis all over the world. Despite this, these non-enveloped RNA viruses are poorly characterized due to the lack of robust and widely available HuNoV culture systems. The two published systems (B cell line and stem cell-derived enteroids) support replication of HuNoVs but the levels of replication are not sufficient for the generation of highly purified virus stocks or the development of culture-based quantification assays. Therefore, improvement of HuNoV in vitro replication is still needed. Murine norovirus and other caliciviruses have provided insights into norovirus replication that paved the way for the development of the current HuNoV culture systems and may also aid in the improvement of these systems. This review will highlight ways in which previous research guided and impacted the development of HuNoV culture systems and discuss ways in which more recent discoveries might be utilized to improve the quality of the HuNoV in vitro replication.

## 1. Introduction

Human noroviruses (HuNoVs) are one of the most common causes of gastroenteritis. These viruses are responsible for ~200,000 deaths in children below five years of age in developing countries and are estimated to cause 685 million infections globally on an annual basis [1,2]. Along with inducing morbidity and mortality by diarrhea, these viruses also have a massive economic impact resulting in approximately $60 billion each year in healthcare costs and missed worker productivity in the U.S. alone [3,4]. Human noroviruses are second only to rotavirus, which annually causes death by dehydration during gastroenteritis in about 215,000 children below the age of five around the world [5]. The introduction of a live, attenuated rotavirus vaccine in 2006 played a pivotal role in diminishing the disease burden of this pathogen [6,7], especially in developed countries, leading to human norovirus becoming a more dominant cause of gastrointestinal illness due to the absence of licensed norovirus vaccines or therapeutics [8,9,10].

Despite the discovery of Norwalk virus in 1972 and the global impact of human norovirus infections, the development of the first in vitro cell culture systems for this pathogen took over 40 years [11,12]. The lack of multigenerational passaging systems in HuNoV has led to a scant understanding of infection mechanisms and prevented traditional methods of vaccine development and specifically the development of live, attenuated vaccines [13]. The research that led to the successful development of the first-generation culture systems for HuNoV was guided by key discoveries made in related viral pathogens. Likewise, improvements in the currently available HuNoV culture systems will likely spring from the increasingly collaborative nature of scientific research and the joint efforts of researchers in multiple fields.

The currently available HuNoV culture systems each utilize a different cell type that supports replication of the virus. The first system to be published uses a transformed B cell line (BJAB) for viral replication, and the second system employs intestinal epithelial cells in the form of stem cell-derived enteroids [12,14]. While the development of these systems has been an exciting breakthrough in the norovirus field, there is still a need to improve in vitro replication of these viruses. A primary area of needed improvement is the viral titers produced by both cell types. Other cultivable caliciviruses typically yield 100,000–1,000,000-fold increases in viral titer, while the B cell and enteroid systems produce 30–100 fold or 30–1000 fold increases, respectively [12,14,15,16,17]. Extensive passaging of HuNoV is also not yet possible with either of the published systems, which, at most, support viral replication out to the fourth passage [12,14]. The inability to generate highly concentrated viral stocks as well as lab-adapted HuNoV strains would provide a significant advancement in the ability to research viral pathogenesis as well as aid in the development of antiviral drugs and vaccines. 

Human noroviruses belong to the family Caliciviridae and several of the cultivable viruses within this family have provided valuable insights for the development of first-generation HuNoV systems. The genus *Norovirus* is subdivided into seven genogroups known to infect human, murine, canine, feline, and bovine host species among others [18]. Human noroviruses are highly diverse and categorized into three genogroups: GI (9 genotypes), GII (20 genotypes), GIV (1 genotype). Strains of the GII.4 genotype have been the most common cause of norovirus-induced gastroenteritis worldwide, however, in recent years we have seen the emergence of GII.17 and GII.2 strains to cause the majority of norovirus illness [19]. Porcine enteric caliciviruses (PEC), which are sapoviruses, have aided in the development of HuNoV cultivation in epithelial cells, while murine norovirus (MNV), has been crucial in the development of the B cell system. 

The genetic diversity of HuNoVs is also reflected in cellular interactions at the molecular level. A cellular factor recognized to play a fundamental role in HuNoV infection is histo-blood group antigen (HBGA). HBGAs are carbohydrates containing structurally related saccharides and are expressed as free oligosaccharide in biological fluids like milk and saliva and linked to proteins or lipids on the surface of red blood cells and mucosal epithelial cells [20,21,22]. The binding of norovirus to HBGAs is strain specific and depends on the type of oligosaccharide epitope expressed on host cells, and infectivity of specific viral strains is dependent on the HBGA type of the host [22,23,24]. These carbohydrates are necessary for replication in both in vitro culture systems, although the way in which they facilitate infection may differ [12,14]. 

## 2. Finding a Permissive Cell Type

Much of the early work pioneering in vitro growth for HuNoVs focused on investigating the ability of intestinal epithelial cells to replicate the virus. The historical paradigm for cellular tropism of enteric viral infection was based on the symptoms of gastrointestinal pathogens. An infected person experiences temporary stomach and intestinal inflammation, causing pain, dehydration, diarrhea, vomiting, and fatigue. So, naturally, it was hypothesized that norovirus would infect intestinal epithelial cells and release pro-inflammatory cytokines involved in inflammation. Intestinal epithelial cells are targeted by several enteric viral pathogens including poliovirus and rotavirus [7,25]. Indeed, human norovirus tropism for epithelial cells was also supported by in vivo data from intestinal sections of acutely infected animals. However, while the virus was detected in epithelial cells, the paucity of the signal was puzzling given the high viral titers shed during symptomatic infection [26,27,28]. 

In 2003, murine norovirus (MNV) was discovered, and it was quickly identified that this virus replicated in dendritic cells and macrophages [17,29]. These studies were the first to prove that a norovirus could target immune cells both in vitro and in vivo. Since its discovery, MNV has become a widely used surrogate for HuNoVs based on its similar shape, size (28–35 nm), genomic organization, and its use in a small animal model [30,31,32,33,34]. In addition, both MNV and HuNoV are spread by fecal-oral transmission, infect the gastrointestinal tract, and are shed in high titers by their respective hosts [35,36,37,38]. The MNV model system has allowed the opportunity to harness the relationship between norovirus replication in vitro and host pathogenesis and immune responses in vivo. It should be noted that the current MNV model is not a perfect replica of HuNoV. For example, mice cannot recapitulate vomiting symptoms of norovirus infection as they do not have emetic reflex, however, gastric bloating, which is known to induce vomiting, is observed in mice and can be used as a pathological substitute [37]. Another difference between HuNoV and MNV infections is the spread of the murine virus to extra-intestinal sites in the mouse, like the spleen, which has not been observed in HuNoV infection [16,37].

Further work investigating the tissue tropism of MNV infection found virus-positive cells in the B cell zones of Peyer’s patches of signal transducer and activator of transcription 1 (STAT-1) deficient and interleukin 10 (IL-10) deficient mice [38,40]. This work was the first to point to B cells as a potential target for MNV. Additional studies revealed diminished viral titers in MNV-infected *Rag1^–/–^* and B cell-deficient mice as compared to wild type mice, which further bolstered the B cell tropism hypothesis and indicated that T cells may also be a potential in vivo target for this virus [16]. More recently, in-depth studies evaluating in vivo cellular tropism of MNV in immunocompetent mice have been conducted and revealed that B cells and T cells, along with macrophages and dendritic cells, are not only infected but actively support norovirus replication in the gut-associated lymphoid tissue (GALT) [36]. Investigations into the in vivo cellular tropism of HuNoVs have revealed these viruses are also able to heavily infect immune cells in chronically infected immunocompromised individuals. Specifically, T cells were overwhelmingly infected by HuNoV compared to the other cell types examined, and this study was the first to demonstrate T cells as a biological target of HuNoVs [41]. Although the presence of non-structural protein was not investigated for infected T cells specifically, the lamina propria contained large regions that were positive for nonstructural proteins indicating the presence of many immune cells supporting viral replication [41]. This HuNoV-positive T cell data is also consistent with MNV tropism data in acutely infected, immunocompetent mice, which show T cells as the predominant cell type infected by the virus [36,41]. It should be noted that B cell infection by HuNoV was not detected in biopsies from immunocompromised individuals, however, B cells were also not found in several of the biopsy samples suggesting that the immunocompromised state of the individuals may have influenced the availability of B cells for infection in those patients [41]. Interestingly, a reduction of HuNoV titers occurs in B cell-deficient individuals as compared to immunocompetent individuals, which phenotypically mimics what is observed in B cell-deficient mice upon infection with MNV [16,42]. Given that these viruses target multiple cell types upon infection, it is anticipated that viral titers would be reduced when a cellular target is not present. It is also important to note that experimental conditions (e.g., immune competent vs. immunocompromised hosts, acute vs. persistent infection) vary widely among these studies, and further work is needed to definitively determine the cellular tropism of HuNoVs in immunocompetent hosts during acute infection. However, collectively, these data demonstrate that noroviruses infect both immune cells and epithelial cells under certain conditions (Table 1 and Table 2).

The early discoveries in MNV immune cell tropism led to work in HuNoVs to determine whether immune cells were permissive to infection in vitro. Initial studies examined blood-derived macrophages and dendritic cells since cell lines from these cell types were shown to replicate MNV to high titers within 24 hours [17]. However, HuNoV replication was not observed [17,44]. Studies examining HuNoV infection in chimpanzees were the first to indicate that cell types beyond epithelial cells were permissive to infection by the human pathogen and revealed that duodenal B cells and dendritic cells were positive for HuNoV capsid proteins [43]. This led to additional investigations on whether HuNoVs were capable of infecting immune cells in vitro. In 2014, it was revealed that both human and murine noroviruses replicated in immortalized B cell lines in vitro (Table 3) and marked the first time HuNoVs had been grown in culture [12,15]. Specifically, infection of B cells with stool samples containing HuNoV resulted in significant increases in both the viral genome (30–100 fold) and synthesis of the major capsid protein after five days of infection [12]. 

**Figure 1:**
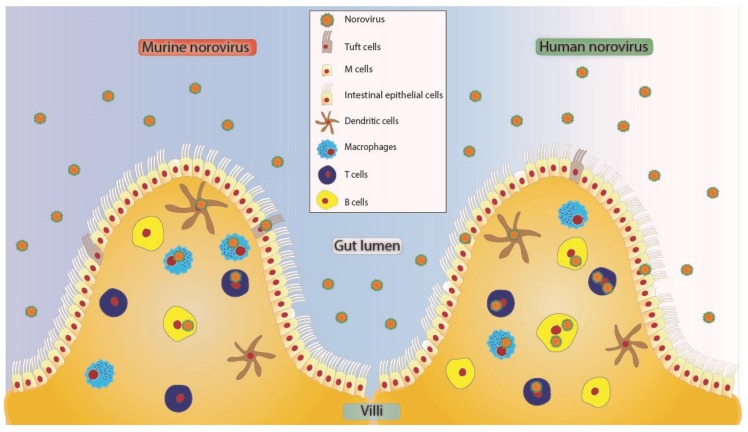
Proposed tropism model of MNV and HuNoV to various cells in the human small intestine [36,50]. These viruses may enter in free form or as clusters [51] potentially crossing through microfold (M) cells [52,53] or through intestinal epithelial cells of the villus. Tuft cells express CD300lf and have been shown to be infected by MNV [39]. Norovirus can further cross the brush border of epithelial cells to infect cells of hematopoietic origin like dendritic cells [36,43], T cells [14,36,41], B cells [36,43], and macrophages [14,36,41]. All the cell types depicted as HuNoV positive have been shown to be positive for viral antigen or genome in either animal studies or human studies.

While the success in cultivating HuNoV in immune cells was groundbreaking, it did not preclude the ability of the virus to replicate in epithelial cells. Similar to studies investigating immune cell infection, early attempts at cultivating HuNoVs in epithelial cells lines were unsuccessful despite exhaustive screening of several primary and immortalized cell types [45]. The invention of organoid systems opened a new avenue of research for cultivating HuNoVs in epithelial cells. Initial attempts to cultivate the virus in 3D-organoid systems were unsuccessful [46], however, in 2016 multiple GII HuNoV strains successfully replicated in human intestinal enteroid (HIE) monolayers derived from stem cells [14]. HIE is a differentiated but non-transformed cell culture consisting of enterocytes, enteroendocrine cells, goblet cells, and Paneth cells, hence forming a replica of the human intestinal microenvironment [47,48]. Although enteroids are different from organoids in that enteriods are not three-dimensional structures derived from primary tissues and grown “organ-like” in an artificial niche, they are a step closer to mimicking the gut or intestinal microenvironment in vitro for the purposes of understanding norovirus propagation in vivo [49]. Successful culture of HuNoVs in the HIE monolayers sets the foundation for the development of more complex in vitro systems containing both epithelial and immune cells to more closely recapitulate the intestinal environment. It has also been recently demonstrated that, during persistent infection, MNV infects specialized epithelial cells, called tuft cells [39]. Interestingly, the investigation of MNV acute infection in vivo has not shown substantial epithelial cell infection. These data are consistent with HuNoV tissue tropism data, where epithelial cell infection has been observed in chronically infected hosts but has not been widely seen in acutely infected individuals [41]. Together these data may point to a disparity in norovirus cell tropism in acute vs. persistent (or chronic) infection [36]. The dual tropism of noroviruses has been recently reviewed [50] and so will not be expanded upon in further detail in this article except to point out that both HuNoV and MNV have been shown to infect a variety of cells types (Figure 1), but the studies publishing this information have varied widely in the conditions used. Therefore, further work is required to determine the cell types targeted by these viruses under varying conditions of host immune status and times of infection. 

## 3. Discovering Cofactors Needed for In Vitro Replication

The discovery of immune cell tropism in murine viruses aided in the development of the first human norovirus culture system. Similarly, the need for and inclusion of cofactors in HuNoV cultivation has also been supported by work from other viruses. For example, it was demonstrated that commensal bacteria enhanced replication of poliovirus [54]. Specifically, the bacterial lipopolysaccharide, which is expressed on the surface of gram-negative bacteria, was specifically responsible for the enhancement of viral infection [54]. As mentioned above, HuNoV infection is dependent on the expression of HBGAs. HBGA-like structures are expressed on the surface of bacterial cells [55], and it has been demonstrated that an HBGA-producing commensal bacterium (*Enterobacter cloacae*) could be bound by HuNoV virus-like particles [56]. These findings led to the discovery that in vitro replication of HuNoVs in B cells is facilitated by the presence of HBGA-expressing commensal bacteria [12,15]. 

Like B cell infection, infection of HIEs requires the addition of a cofactor, at least for some strains of human norovirus [14]. PEC causes diarrhea in gnotobiotic and weaned pigs [57,58]. Struggles to cultivate this virus in vitro eventually led to the discovery that intestinal contents, and specifically bile acids, were key to successful laboratory replication of the virus [59,60,61]. In PEC, bile acids act by downregulating type I Interferon (IFN)-mediated signal transduction and facilitating release from endosomes into the cell cytoplasm, which allowed for PEC replication in LLC-PK cells [59,62,63]. It has also recently been shown that specific mutations in lab-adapted strains of PEC allow for improved replication in vitro and investigation of similar residues in HuNoV may also aid in improving its replication in vitro [64]. Given the key role intestinal contents and bile acids played in the successful cultivation of PEC, this compound was also examined for involvement in HuNoV replication. Initial studies examining HuNoV replication in epithelial cells lines found that simple addition of bile acids to these cells did not result in HuNoV amplification [45]. However, upon examination of the role of bile acids in HuNoV replication in HIEs, it was found that replication of some viral strains was dependent on (or enhanced by) non-toxic levels of bile acids. Like PEC, it is suggested that the increase in HuNoV replication in the presence of bile acids is due to the effect of bile on the cells and not on the virus [14,65]. However, X-ray crystallography has shown that bile acids bind at partially conserved pockets on the P domain of some genotypes and stabilize P domain loops in HBGA non-binders. Moreover, bile acids augmented binding of GII.10 with HGBA [65]. Similarly, in MNV, bile acids have been demonstrated to bind at the interface of the P1 and P2 domains of the capsid and enhance interaction with its cellular receptor [66]. Both of these observations point to an impact of bile acids on the viral particle itself. Bile carries out multiple cellular functions such as facilitating digestion and absorption of fat, acting as a detergent, and regulating cell metabolism and inflammation, some or all of which may play a role in HuNoV infection [67]. Furthermore, bile acids are also acted upon and modified by commensal bacteria in the small intestine [68,69]. Although bacteria are not involved in HuNoV replication in HIEs, given the demonstrated role of commensal bacteria in norovirus infection, it may be that commensal bacteria influence in vivo infection through both direct and indirect mechanisms [12]. 

While discoveries in other viruses have aided in the development of HuNoV culture systems, not all research has proven to be beneficial in the cultivation of this pathogen. For example, early methods of astrovirus cultivation began with the generation of cell culture-adapted isolates using primary cells and treatment with trypsin that allowed for serial propagation of the virus in tissue culture [70,71]. Trypsin has also been shown to enhance the infectivity of rotavirus by facilitating cell entry [72,73,74]. However, the addition of trypsin did not result in the successful cultivation of HuNoVs in transformed epithelial cells lines [45]. Nevertheless, the advancement of research in related enteric RNA viruses has greatly assisted the growth of techniques and ideas in the norovirus field and led to the development of the current cultivation systems. 

## 4. Improvement of Current Models or the Discovery of New Ones

More recent discoveries in the norovirus field may hold the key to improving the current methods of in vitro cultivation of HuNoV. One such discovery was the identification of CD300lf as the receptor for MNV [75,76]. CD300lf is an immune-regulatory protein present on the hematopoietic cell (especially myeloid cells) and tuft cell surface [39,66,76]. MNV interacts with CD300lf and further engages CD300lf-like host ligands through multiple binding sites [66]. It was also demonstrated that cells that were refractory to infection by MNV could support infection if they were modified to express the CD300lf receptor [75]. The proteinaceous receptor required for HuNoV infection of immune and epithelial cells is not yet known, but given the important role MNV has played in the advancement of our understanding of HuNoV biology, it is likely that discovery of the MNV receptor will open new doors for the identification of the cellular receptor for HuNoV, which may aid in increasing viral replication in vitro. 

Alongside the discovery of the cellular receptor for MNV was the discovery of a distinct cell type that is infected by the virus during persistent infection. These cells were called brush, multi-vesicular, fibrillovesicular, caveolated, or tuft cells [39]. Tuft cells express CD300lf and are targeted by MNV in the mouse intestine during persistent infection. Identification of this persistently infected cell type could lead to its use in cell culture systems. Specifically, persistent in vitro infection could be used for generation of lab-adapted or attenuated norovirus strains, which would aid in vaccine development. 

Another novel development in norovirus biology is the role of exosomes in viral egress [51]. Non-enveloped viruses are classically thought to escape from infected cells through cellular lysis, however, over the last several years, multiple research groups have shown that cell destruction is not always necessary for virus spread [77]. For example, poliovirus can release viral antigens into the media without the death of human neuroblastoma or K562 cells [78,79]. Similarly, hepatitis A and hepatitis E viruses were shown to be masked in a membrane while circulating in the bloodstream [80,81]. These observations led to the hypothesis that non-enveloped viruses have alternate mechanisms of egress besides cell lysis. Supporting this hypothesis, several enteroviruses including Coxsackievirus, poliovirus, and rhinovirus have been found to form viral clusters inside extracellular vesicles (EVs) in vitro [82,83,84,85]. EVs are membrane-bound vesicles secreted by living or dead cells, which carry peptides, microRNAs, or other cargo such as viruses or pathogenic molecules [82,86]. Recently, it was shown that MNV-1 and HuNoV can exit cells cloaked in EVs [51]. These virus-containing vesicles are shed in the stool of infected humans/mice and remain intact during fecal–oral transmission [51]. Moreover, for MNV, it was found that these virus-containing EVs still required the CD300lf receptor for the infection of new cells [51]. Given that exosomes also play an important role in cellular communication, the impact of viral infection on this cellular pathway may lead to new discoveries involving antiviral immune responses and may also be key in identifying factors responsible for low levels of viral replication in vitro [87,88]. 

**Figure 2:**
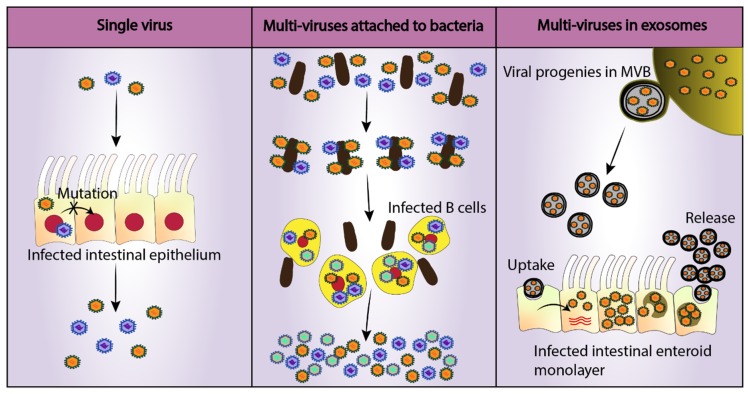
Viral mechanisms to increase the cellular multiplicity of infection (MOI). Single RNA viruses lack proofreading mechanisms diminishing the ability of the virus to undergo successful replication in its host due to the production of attenuating mutations [89]. On the other hand, infection of a single cell with multiple viruses increases infection efficiency and viral fitness [51]. Two such mechanisms have been identified in enteric viruses: Attachment to commensal bacteria (poliovirus) and egress in extracellular vesicles (rotavirus) [51,90]. A similar mechanism may be employed by HuNoV, which could explain enhanced B cell replication in vitro in the presence of enteric bacteria such as *Enterobacter cloacae* [12]. Noroviruses are also found inside multi-vesicular body (MVB) derived exosomes and cause higher infection as compared to single viruses of same MOI [51].

Exosomes are not only involved in viral egress but also appear to facilitate increased virus production by infected cells [51]. RNA viruses are notorious for the lack of proofreading mechanisms in their polymerases resulting in a high mutation rate in a range of 10^−3^–10^−5^ mutations per nucleotide copied, as estimated by independent biochemical and genetic approaches [89]. This is almost a million-fold higher as compared to mutation rates of cellular DNA, indicating that there are rarely identical copies of each virus [89]. This suggests that any single progeny is most likely not able to undertake a successful replication cycle in the next host. Each virus may have multiple attenuating mutations and thus require the presence of other viral progeny with potentially compensating mutations. This idea challenges the “free independent virus particle” idea of viral transmission and is increasingly supported biologically with the discovery of mechanisms by which multiple viral particles can infect the same cell simultaneously. One example is the cellular escape of multiple viral particles within a single exosome (mentioned above). Exosomes containing noroviruses can carry around one to five viruses per vesicles and are more infectious than individual viruses or free viral particles [51]. When examined directly, infection by EV-encased viruses resulted in a higher number of progeny viruses produced compared to infection with “free” virus suggesting there are manifold barriers to viral entry when the number of viruses bound to cells are low [82,83,84,85]. A different mechanism yielding a similar overall result can be seen with the use of bacteria by poliovirus (Figure 2). It was demonstrated that attachment of polioviruses to enteric bacterium enhanced both viral infection efficiency and recombination leading to increased fitness of viral progeny [90]. Enhanced infection of HuNoV in B cells in the presence of bacteria may be the result of an analogous mechanism or it may indicate an alternate role for HBGAs as a means of viral aggregation that improves viral infection. Ultimately, when viral particles enter in high concentrations such as on bacteria or inside EVs, the probability of viral replication, translation of functional proteins, and sharing genomic or replication machinery increases, potentially giving rise to greater genetic diversity in progeny viruses and improved viral fitness [90,91,92]. Therefore, research investigating mechanisms of viral aggregation may also lead to discoveries that improve HuNoV output in cell culture systems.

While improving replication in the currently available B cell and HIE systems may be the most straightforward path to improving laboratory cultivation of HuNoV, other avenues remain. One such method would use the humanized mouse model that has been developed for HuNoV. These mice were engrafted with human CD34^+^ hematopoietic stem cells, to test the importance of human immune cells for infection [93,94]. Interestingly, the presence of HuNoV structural and non-structural proteins was detected in cells in both humanized and non-humanized immune-deficient mice [94]. This surprising observation may be useful in isolation and culture of lymphocytes from model systems that could serve as a foundation for a new and novel in vitro culture system for HuNoV. 

## 5. Conclusions

Considering the economic and health burdens caused by HuNoV, a better understanding of virus-host interactions through improved cell culture models is the need of the hour. Use of the currently available B cell and HIE systems has already led to their use in the testing of antiviral compounds and facilitated studies on virus inactivation, respectively [95,96]. Additionally, HIEs allowed for the implementation of true HuNoV neutralization assays rather than using HBGA binding assays as a surrogate for neutralization [97]. However, restricted viral replication in both systems limits their use in a detailed examination of viral infection and pathogenesis. Detailed cellular and genetic studies would ultimately give rise to the discovery of attachment and/or entry receptor proteins as well as identify antiviral mechanisms that suppress HuNoV replication in specific cell types. Furthermore, improved in vitro replication and passaging of generated viruses would allow for the creation of lab-adapted viruses and the development of live, attenuated HuNoV vaccines. Improved in vitro replication will also allow for increased crossover between virology and immunology, to discover answers to other characteristics of HuNoV infection, such as the lack of long-term protective immunity generated from viral infection. The progress made thus far in the development of HuNoV in vitro culture systems has been bolstered by knowledge gained from research on surrogate viruses, which is a testament to the collaborative nature of science, and future improvements to these systems may also come from discoveries made in other viruses. 

## Figures and Tables

**Table 1:** In vivo cellular tropism of murine norovirus (MNV).

Cell Type	Immunocompetent Host		Immunocompromised Host	
	Acute ^1^	Persistent	Acute	Persistent
Epithelial cells	+ ^2^	– ^3^	+ ^4^	N.D.
Tuft cells	N.D.	+ ^3^	N.D.	N.D.
Macrophages	+++ ^2^	N.D.	+++ ^4, 5^	N.D.
Dendritic cells	++ ^2^	N.D.	+++ ^4, 5^	N.D.
B cells	+ ^2^	N.D.	N.D.	N.D.
T cells	+++ ^2^	N.D.	N.D.	N.D.

*Notes:*^1^ N.D. = Not done. ^2^ Stained for viral antigen and replication intermediate [36]. ^3^ Stained for viral non-structural proteins [39]. ^4^ Stained replication intermediate in epithelial cells and lamina propria [29,37]. ^5^ Stained viral antigen [29].

**Table 2:** In vivo cellular tropism of human norovirus (HuNoV).

Cell Type	Immunocompetent Host		Immunocompromised Host	
	Acute ^1^	Persistent ^1^	Acute ^1^	Persistent ^1^
Epithelial cells	N.D.	N.D.	+ ^3^	++ ^4^
Tuft cells	N.D.	N.D.	N.D.	N.D.
Macrophages	N.D.	N.D.	N.D.	+ ^5^
Dendritic cells	+ ^2^	N.D.	N.D.	+ ^5^
B cells	+ ^2^	N.D.	N.D.	– ^5^
T cells	N.D.	N.D.	N.D.	+++ ^5^

*Notes:*^1^ N.D. = not done. ^2^ Detected viral antigen in tissue section of chimpanzees [43]. ^3^ Detected viral antigen in tissue sections of gnotobiotic pigs [27,28]. ^4^ Detected viral antigen and non-structural proteins [41]. ^5^ Stained for viral antigen in cells and non-structural proteins detected in the lamina propria [41].

**Table 3:** Summary of cell types which support in vitro replication of HuNoV and MNV.

Cell Type	HuNoV	MNV
Epithelial cells	Yes	No
Macrophages	No	Yes
Dendritic cells	No	Yes
B cells	Yes	Yes
